# Analgesic and Anti-Arthritic Potential of Methanolic Extract and Palmatine Obtained from *Annona squamosa* Leaves

**DOI:** 10.3390/ph17101331

**Published:** 2024-10-05

**Authors:** Caren Naomi Aguero Ito, Elisangela dos Santos Procopio, Natália de Matos Balsalobre, Lucas Luiz Machado, Saulo Euclides Silva-Filho, Taíse Fonseca Pedroso, Caroline Caramano de Lourenço, Rodrigo Juliano Oliveira, Arielle Cristina Arena, Marcos José Salvador, Cândida Aparecida Leite Kassuya

**Affiliations:** 1 Health Sciences College, Federal University of Grande Dourados (UFGD), Dourados 79804-970, MS, Brazil; itonaomi@live.com (C.N.A.I.); elisangelaprocopiosan@gmail.com (E.d.S.P.); nataliabalsalobre@hotmail.com (N.d.M.B.); 2 Pharmaceutical Sciences, Food and Nutrition College, Federal University of Mato Grosso do Sul (UFMS), Campo Grande 79070-900, MS, Brazil; lucaslmachado11@gmail.com; 3 Institute of Biology, Department of Plant Biology, University of Campinas (UNICAMP), Campinas 13083-862, SP, Brazil; taisefpedroso@gmail.com (T.F.P.); ca_jau@hotmail.com (C.C.d.L.); marcosjs@unicamp.br (M.J.S.); 4 Stem Cell, Cell Therapy and Toxicological Genetics Research Centre (CeTroGen), Medical School, Federal University of Mato Grosso do Sul (UFMS), Campo Grande 79070-900, MS, Brazil; rodrigo.oliveira@ufms.br; 5 Institute of Biosciences of Botucatu, Department of Structural and Functional Biology, São Paulo State University (UNESP), Botucatu 18618-970, SP, Brazil; arielle.arena@unesp.br

**Keywords:** *Annona*, alkaloids, anti-hyperalgesic, arthritis, efficiency, inflammation

## Abstract

**Background/Objectives**: *Annona squamosa* is used in folk medicine to treat pain and arthritis. Palmatine is an alkaloid isolated from several plants, including *A. squamosa* leaves. The aim of the present study was to investigate the analgesic, anti-arthritic, and anti-inflammatory potential of the methanolic extract of *A. squamosa* (EMAS) and palmatine. **Methods**: The chemical profile of EMAS was evaluated by ultra high-performance liquid chromatography with electrospray ionization coupled to mass spectrometry (UHPLC-ESI/MS). EMAS and palmatine were evaluated in carrageenan-induced pleurisy, zymosan-induced joint inflammation, formalin-induced nociception, and tumor necrosis factor (TNF)-induced mechanical hyperalgesia in experimental models in mice. A cytotoxicity test of EMAS and palmatine was performed using a methylthiazolidiphenyl-tetrazolium (MTT) bromide assay. **Results**: The analysis of the chemical profile of the extract showed the presence of palmatine, liriodenine, and anonaine. Oral administration of EMAS and palmatine significantly reduced leukocyte migration and oxide nitric production in the carrageenan-induced pleurisy model. EMAS and palmatine reduced mechanical hyperalgesia, leukocyte migration, and edema formation in the joint inflammation induced by zymosan. In the formalin test, palmatine was effective against the second-phase nociceptive response, mechanical hyperalgesia, and cold allodynia. In addition, palmatine reduced mechanical hyperalgesia induced by TNF. EMAS and palmatine did not demonstrate cytotoxicity. **Conclusions**: The present study showed that *A. squamosa* and palmatine are analgesic and anti-inflammatory agents, and that the anti-hyperalgesic properties of palmatine may involve the TNF pathway. Palmatine may be one of the compounds responsible for the anti-hyperalgesic and/or anti-arthritic properties of this medicinal plant.

## 1. Introduction

Inflammation is a complex process involving multiple signaling pathways that, if unsolved, can lead to a chronic inflammatory process [1]. Neutrophils are normally the first responders (among others) to acute inflammation, and they contribute to the release of inflammatory (prostaglandins (PGs), leukotrienes, cytokines, chemokines) and pro-resolving mediators [2]. When this process is not healed, the subacute phase can occur. In this phase, neutrophils release lysosomal enzymes, which increase capillary permeability and cause tissue destruction, and monocytes (macrophages) act as cellular defense cells. Failure to resolve inflammatory processes can lead to increased cells and several inflammatory mediators, resulting in chronic inflammation [2,3]. In chronic inflammation, pain, edema, and loss of tissue function are three of the five cardinal signs that [4] may be present [5].

The main pharmacological treatments of pain are opioid analgesics and nonsteroidal anti-inflammatory drugs (NSAIDs), whose long-term use can cause various side effects. These adverse effects include opioid-induced dependence, NSAID-induced ulcers (non-selective NSAIDS), and/or cardiovascular thrombotic events (selective cyclooxygenase(COX)-2 inhibitors) [6,7]. The main drugs used to treat inflammation are NSAIDs and glucocorticoids analogs. Chronic treatment with the latter can lead to adrenal insufficiency, insulin resistance, and other health problems [8,9]. Considering the need for new anti-inflammatory and analgesic drugs, analysis of the biological activities of medicinal plants and their isolated compounds is important for the development and discovery of new drugs.

*Annona squamosa* L. (Annonaceae) is an exotic tree, known as “fruta-do-conde” (Brazil) [10] or Seetha pazham [11], popularly used by oral route for urinary disorders (Philippines) [12], hair growth (India) [13], inflammation, and cancer, among others [11,14,15]. In the Philippines, it is popularly used topically for headache and mumps, cuts, and wounds [12,16]. *A. squamosa* can be found in various tropical and subtropical regions of the world, especially in the American continent [17]. Several states in Brazil cultivate this plant, which is known for its fruit. The trees grow from 3 m to 8 m high, with large branches, brownish bark, and thin leaves [18].

The juice of the whole plant or the powder of the leaves are used for body aches [13,19] and arthritis [20]. The decoction of the leaves is widely used in folk medicine in China to treat lymphadenopathy [14] and in Brazil, after preparation by maceration, infusion, or decoction, the leaves are used to treat hemorrhoids, labyrinthitis, digestive, and rheumatic disorders [17,21,22]. In scientific studies, the ethanolic extract obtained from the leaves of *A. squamosa* have shown efficacy in the acetic acid-induced nociception model and in the hot plate test [23]. In addition, an ethanolic extract of the leaves and seeds of *A. squamosa* reduced carrageenan-induced paw edema [23,24]. Caryophyllene oxide was isolated from petroleum ether extract of the bark of *A. squamosa*, and it seems to be the compound responsible for several biological properties [25]. Palmatine has several pharmacological properties such as hypolipidemic action [26], antiproliferative [27], gastroprotective [28], neuroprotective [29], antidepressant [30], chondroprotective [31], anti-inflammatory [32], antibacterial, antiviral, and hepatoprotective activities [33,34], and alleviating lung injury [35], among others. In addition, palmatine showed anti-hyperalgesic effects against diabetic neuropathy (mechanical and thermal hyperalgesia) [30] and trigeminal neuralgia models (mechanical hyperalgesia) in rats [36]. No studies have shown the anti-nociceptive action of palmatine.

In view of this, the present research evaluated the presence of alkaloids and the chemical profile of the methanolic extract of *A. squamosa* leaves (EMAS). The biological properties (anti-inflammatory, anti-nociceptive, and anti-hyperalgesic) of EMAS and its isolated compound (palmatine) were analyzed in models of nociception (formalin), paw (carrageenan), and articular (zymosan) inflammation as well as its cytotoxic activity. The mechanisms of action of palmatine were also investigated in relation to the tumor necrosis factor (TNF) pathway. This work is relevant since the involvement of palmatine in the analgesic and anti-arthritic responses of *A. squamosa* has not been fully elucidated.

## 2. Results

### 2.1. Chemical Analysis of EMAS and Isolation and Structure Elucidation of Palmatine

**Figure 1:**
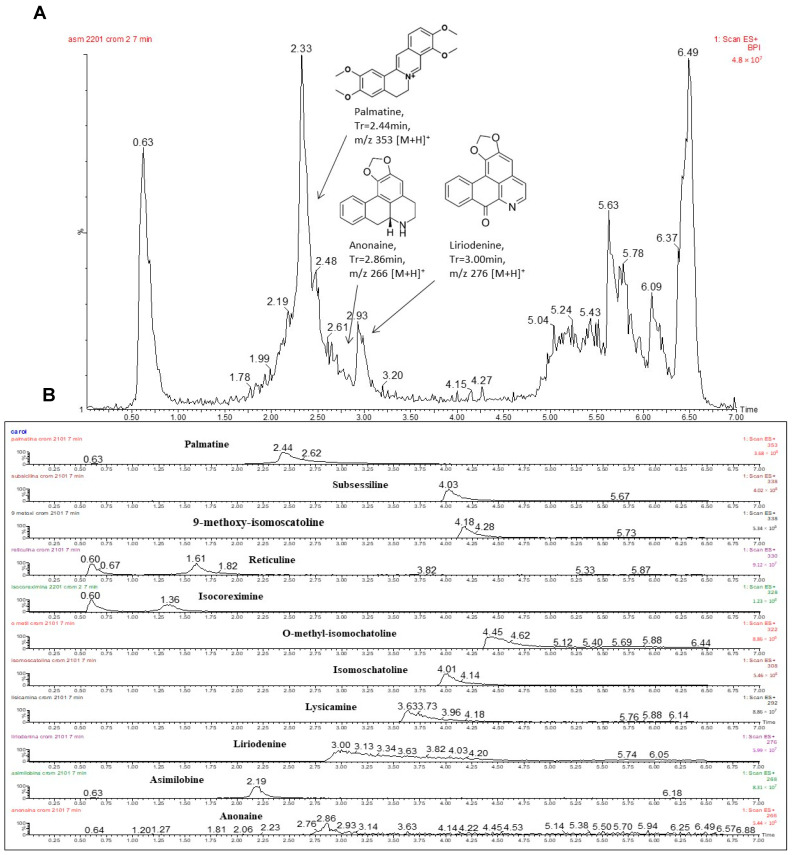
UHPLC-MS chromatograms. (**A**) From the methanolic extract of the leaves of *A. squamosa* (EMAS) with some identified alkaloids. (**B**) From the standard samples of alkaloids used as a standard.

The results of ESI-MS and UHPLC-MS-MS analysis of EMAS allowed for the identification of the alkaloids anonaine, liriodenine, and palmatine (Table 1 and Figure 1). These data are in agreement with the chemical profile presented by the Annona genera and the Annonaceae family. As the main constituents of the *Annona* leaves are alkaloids, for further purification, an acid–basic extraction was performed with EMAS, resulting in the alkaloidal fraction. Fractionation of the alkaloidal fraction resulted in the isolation of palmatine alkaloid. The purity and identification of this substance were confirmed using spectroscopic (UV, IR, 1D, and 2D NMR) and mass spectrometric (ESI-MS) methods, and by comparison with the literature data [37]. The elucidation of the structure of palmatine, with the interpretation of carbon and proton NMR data and a reference article which helped to confirm the structure, is shown in the Supplementary Materials.

### 2.2. Effects of EMAS and Palmatine on Carrageenan-Induced Pleurisy

**Figure 2:**
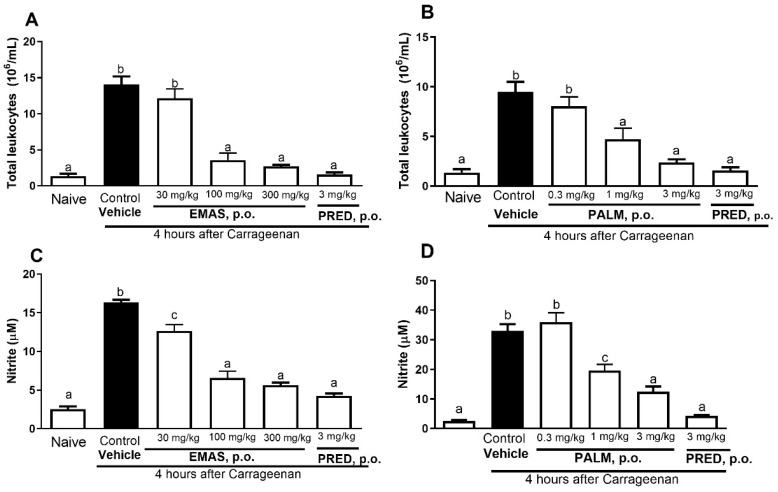
Effects of oral administration of EMAS and palmatine on leukocyte migration (**A**,**B**) and nitric oxide production (**C**,**D**) in pleurisy model induced by carrageenan. Animals received, by gavage, EMAS (30, 100, 300 mg/kg), palmatine (0.3, 1, 3 mg/kg), vehicle (naïve and control groups), or prednisolone (PRED, 3 mg/kg), and, after 1 h, intrathoracic injection of carrageenan was given to groups, except naïve. Each bar represents mean ± SEM of 6 animals. Letters a, b and c indicate significant differences among groups according to Tukey’s multiple comparison test.

Evaluation of pleurisy induced by carrageenan showed a reduction in leukocyte migration of 74% for a dose of 100 mg/kg of EMAS, 81% for a dose of 300 mg/kg of EMAS, 50% for a dose of 1 mg/kg of palmatine, and 75% for a dose of 3 mg/kg of palmatine (Figure 2A,B), when compared to the control group of mice. The oral administration of 30 mg/kg of EMAS and 0.3 mg/kg of palmatine did not interfere with leukocyte migration to the mouse pleural cavity. Furthermore, the groups treated with EMAS (100 and 300 mg/kg) did not differ statistically from the groups treated with the reference drug (prednisolone) or those from the naïve group.

Evaluation of pleurisy induced by carrageenan showed a reduction in leukocyte migration of 22.64% for a dose of 30 mg/kg of EMAS, 59.85% for a dose of 100 mg/kg of EMAS, 65.48% for a dose of 300 mg/kg of EMAS, 40.61% for a dose of 1 mg/kg of palmatine, and 62.42% for a dose of 3 mg/kg of palmatine (Figure 2C,D), when compared to the control group of mice. The oral administration of 0.3 mg/kg of palmatine did not interfere with nitrite production in the pleural cavity. Oral administration of EMAS (Figure 2C) and palmatine (Figure 2D) did not differ statistically from the groups treated with prednisolone or those from the naïve group, when compared to the control group. Moreover, the analysis demonstrated that palmatine could act in a dose-dependent manner. 

### 2.3. Effects of EMAS and Palmatine on Zymosan-Induced Joint Inflammation

**Figure 3:**
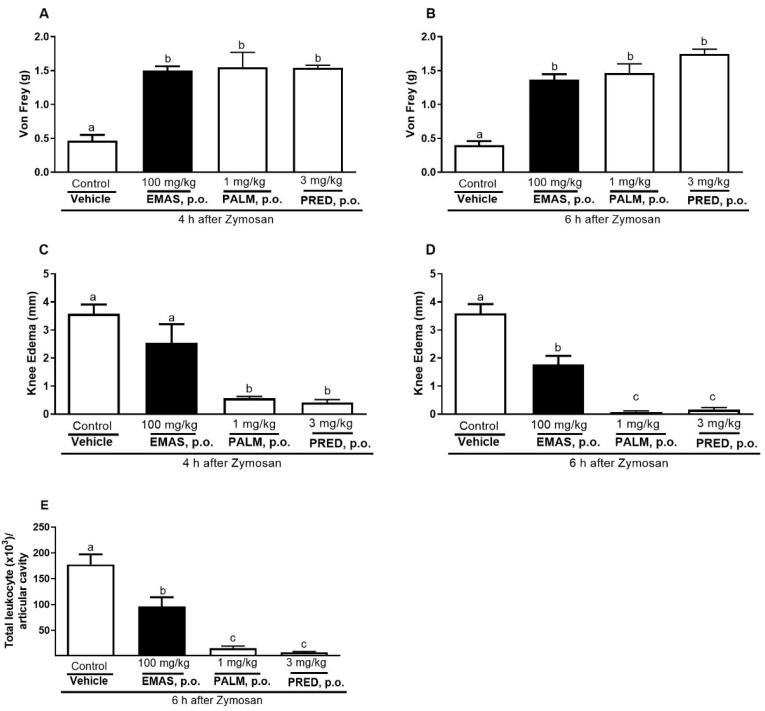
Effects of oral administration of EMAS and palmatine on mechanical hyperalgesia (**A**,**B**), knee edema (**C**,**D**), and on leukocyte migration (**E**) caused by zymosan-induced joint inflammation in female mice. Animals received, by gavage, EMAS (100 mg/kg), palmatine (1 mg/kg), saline solution (control group), or the reference drug prednisolone (PRED, 3 mg/kg) and, after 1 h, 200 μg of zymosan was injected into left knee. After induction of inflammation, mechanical hyperalgesia and edema were measured four (**A**,**C**) and six (**B**,**D**) hours later, respectively. After that, animals were euthanized and joint exudate was used to assess leukocyte migration (**E**). Each bar represents mean ± SEM of 6 animals. Letters a, b, and c indicate significant differences among groups according to Tukey’s multiple comparison test.

After 4 h of zymosan administration in the knee, oral exposure to a dose of 100 mg/kg of EMAS, 1 mg/kg of palmatine, and prednisolone (PRED) significantly inhibited mechanical hyperalgesia in 68, 70, and 70%, respectively. After 6 h of zymosan administration in the knee, the inhibition of mechanical hyperalgesia induced by EMAS was 70% for 100 mg/kg, while for palmatine it was 73%, and for prednisolone (PRED) it was 77% (Figure 3A,B). The results of palmatine- and EMAS-treated groups showed no statistical difference from those treated with prednisolone; however, all these groups differed from the control group (Figure 3A,B).

After 4 h of zymosan administration in the knee, oral exposure to a dose of 1 mg/kg of palmatine, and of prednisolone (PRED) significantly inhibited edema in 84 and 88%, respectively. EMAS did not interfere with the edema at this point. After 6 h of zymosan administration in the knee, oral exposure to a dose of 100 mg/kg of EMAS, 1 mg/kg of palmatine, and of prednisolone (PRED) significantly inhibited edema in 51%, 98%, and 96%, respectively. The results of the palmatine- and prednisolone-treated groups showed no statistical difference between themselves; however, both groups differed from the control group and EMAS-treated group (Figure 3C,D).

Six hours after the induction of joint inflammation by zymosan (Figure 3E), leukocyte recruitment was reduced by the treatment with EMAS (51%), palmatine (91%), and prednisolone (96%). Regarding leukocyte recruitment, the results of the groups treated with palmatine and prednisolone showed no statistical difference between themselves; however, both groups differed from the control group and EMAS-treated group (Figure 3E).

### 2.4. Effects of EMAS and Palmatine on Formalin-Induced Nociception

**Figure 4:**
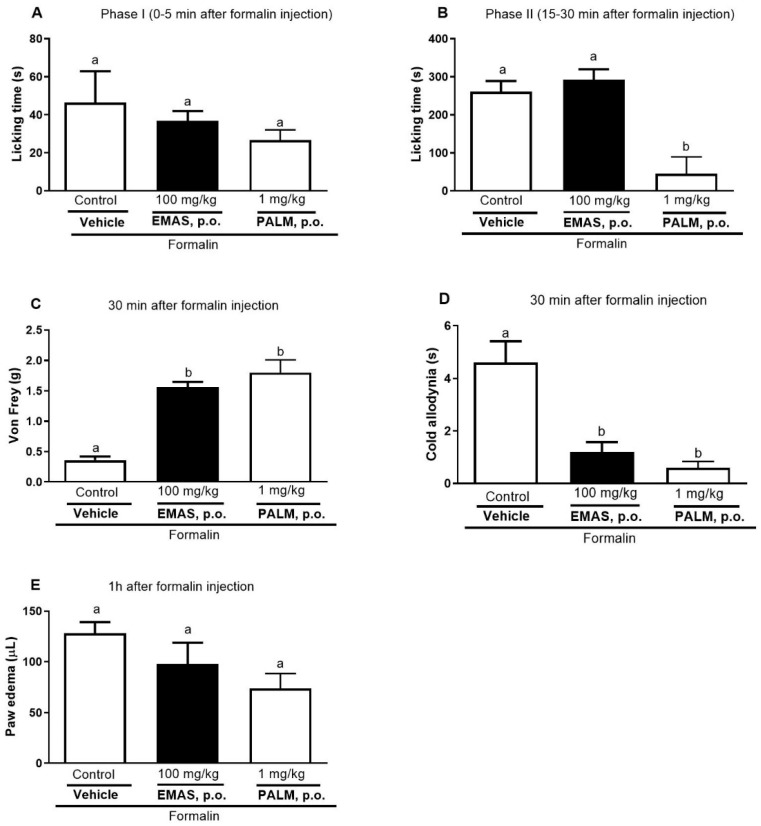
Effects of oral administration of EMAS and palmatine on formalin test. One hour before applying 30 μL of 1% formalin, animals were orally treated with EMAS (100 mg/kg), palmatine (PALM 1 mg/kg), or vehicle (control group). First phase represents neurogenic phase (**A**), while second phase is inflammatory phase (**B**). Paw withdrawal threshold (Von Frey test) (**C**) and cold sensitivity (**D**) were performed immediately after formalin test. Formalin-induced edema was measured one hour after formalin application (**E**). Each bar represents mean ± SEM of 5 animals. Letters a and b indicate significant differences between groups according to Tukey’s multiple comparison test.

Formalin-induced nociception and paw edema were not reduced by EMAS (100 mg/kg) and palmatine (1 mg/kg) in the first phase (Figure 4A,E). In the second phase (inflammatory phase) (Figure 4B), treatment with palmatine significantly reduced the time the animals bit or licked the paw (83%), when compared to the control group.

Furthermore, EMAS (77%) and palmatine (80%) decreased mechanical hyperalgesia (Figure 4C) and reduced cold sensitivity (Figure 4D) by 74% and 87%, respectively, when compared to the control group.

### 2.5. Effect of Palmatine on TNF-Induced Hyperalgesia

**Figure 5:**
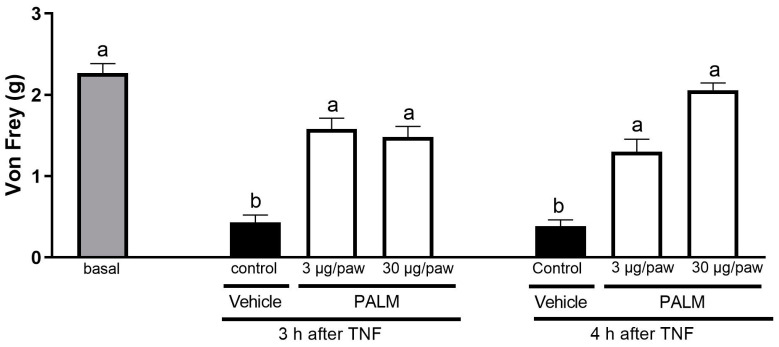
The effects of the intraplantar administration of palmatine on TNF-induced mechanical hyperalgesia. One hour before the injection of 20 μL of TNF into the right hind paw, the animals were intraplantar treated with saline solution or palmatine (PALM 3 and 30 µg/paw) in the same region as the inducing agent. Three and four hours after the TNF injection, mechanical hyperalgesia (Von Frey test) was analyzed. In addition, the basal response to mechanical stimulus was performed before treating the animals. Each bar represents the mean ± SEM of 5 animals. The letters a and b indicate significant differences among groups according to Tukey’s multiple comparison test.

Intraplantar treatment of 3 µg/paw (73% and 71%) and 30 µg/paw (71% and 81%) of palmatine prevented mechanical hyperalgesia 3 and 4 h after TNF compared to the control group (Figure 5). Furthermore, these two doses (3 and 30 µg/paw) of oral palmatine administration, 3 and 4 h after TNF injection, differed from the control group and did not statistically differ from baseline. The baseline values of the three groups were not statistically different.

### 2.6. Effect of EMAS and Palmatine on Leukocyte Viability by MTT

EMAS and palmatine showed no cytotoxicity in vitro. The cell viability of EMAS (96, 93, and 84%) and palmatine (72.89, and 88%) at concentrations of 10, 30, and 90 μg/mL did not induce cytotoxicity (results not shown).

## 3. Discussion

The present research showed the anti-nociceptive and anti-arthritic properties of EMAS and palmatine in experimental models. The anti-inflammatory effects of *A. squamosa* leaves have been extensively explored in the literature [23,38,39]; however, available studies of analgesic [23] and anti-arthritic properties with this plant are scarce. The leaves of *A. squamosa* are used by the population to treat body pain [13,19] and rheumatic disorders [17,20]. To our knowledge, this is the first study to demonstrate that EMAS is effective against painful and inflammatory parameters in zymosan and formalin models. No cytotoxicity was found for EMAS and palmatine in the tests performed in this study, corroborating the studies showing that palmatine is not cytotoxic. Palmatine was isolated from EMAS and the purity of the substance was demonstrated (Supplementary Materials). Palmatine is an alkaloid, with a wide spectrum of its pharmacological effects already described in the literature; however, its anti-nociceptive and anti-arthritic effects have not been totally explored. In the present work, the analgesic and anti-arthritic activities of EMAS seem to be induced by palmatine, and the mechanisms of action could be related to the TNF pathway.

Different parts of *A. squamosa* (bark, leaves, fruit, seeds) have shown substances with pharmacological, nutraceutical, and biological potential [18,40,41]. The peel of its fruit (epicarp), for example, has mostly shown spathulenol, a substance with anti-inflammatory, anti-nociceptive, antimicrobial, and antiproliferative potential [42,43]. In the ethanolic extract of the leaves, sesquiterpenes such as *trans*-caryophyllene have been identified [44]. *Trans*-caryophyllene, for example, reduced mechanical hyperalgesia and thermal nociception in a chronic model of neurogenic pain (sciatic nerve constriction) [45]. *Trans*-caryophyllene reduced mechanical hyperalgesia and IL-6 and IL-1 β levels in a model of chronic diabetic neuropathy induced by streptozotocin [46]. This substance reduced paw inflammation induced by several inflammatory agents [47]. In another study, *trans*-caryophyllene reduced capsaicin-induced nociception [48]. In addition to these, other substances with anti-inflammatory and anti-nociceptive potential present in the leaves of *A. squamosa* have also been identified, such as trans-caryophyllene, phytol, and humulene. *Trans*-caryophyllene (23.0%) and β-elemene were isolated from the essential oil of *A. squamosa* [44,49]. Phytol and trans-caryophyllene have shown thermal (hot plate test) and chemical (formalin, acetic acid) anti-nociceptive effects in acute nociception models [45,50].

The carrageenan-induced pleurisy model in mice is a relevant model to study the anti-inflammatory effects of new drugs in vivo [51]. Pure palmatine and EMAS have been tested in a pleurisy model and the analysis demonstrated that doses of 100 and 300 mg/kg, but not the dose of 30 mg/kg, of EMAS, and that doses of 1 and 3 mg/kg, but not the dose of 0.3 mg/kg, of palmatine are effective, which could indicate that palmatine induced a dose-dependent inhibition (Figure 2A,B). In relation to nitrite production, all the tested doses of EMAS inhibited this parameter, indicating a possible action in oxide nitric production. Doses of 1 and 3 mg/kg, but not a dose of 0.3 mg/kg, of palmatine inhibited nitrite production in pleural exudate, corroborating the findings of Yan et al. [28] that showed the inhibition of the oxide nitric pathway (in vitro) by palmatine. The ethanolic extract obtained from *A. squamosa* leaves also showed an anti-inflammatory property in carrageenan-induced paw edema [23], while palmatine, in an in vitro study, reduced the production of pro-inflammatory mediators (TNF-α, IL-1β, IL-6, and NO) and increased the production of IL-10 [32]. These new results indicate that EMAS and palmatine had anti-inflammatory action in the pleurisy model and showed that palmatine could act in a dose-dependent manner.

Several scientists have classified zymosan-induced joint inflammation in rodents as an arthritis experimental model for drug discovery [52,53]. Zymosan is derived from *Saccharomyces cerevisiae* and is classified as an immunogenic and inflammatory agent. When zymosan is injected into the knee cavity, it can induce hyperalgesia, joint edema, and neutrophil recruitment [53], which can characterize acute inflammatory processes in the knee. In previous studies, palmatine demonstrated chondroprotective effects in an osteoarthritis model [31]. The 100 mg/kg dose of EMAS and the 1 mg/kg dose of palmatine were effective in inhibiting mechanical hyperalgesia, leukocyte migration, and edema in zymosan joint inflammation. These results show that EMAS and palmatine have anti-arthritic potential, which may explain, at least in part, the popular use of *A. squamosa* for the treatment of arthritis.

The formalin test is a nociceptive model used to evaluate new products with analgesic activity in the two phases of pain: neurogenic pain, related to direct stimulation of nociceptor fibers, and inflammatory pain, related to inflammatory mediators [54]. Previous studies have shown that extracts of *A. squamosa* leaves exhibited anti-nociceptive effects in acetic acid (peripheral action) and the hot plate test (central action) [23], while seed extract showed anti-edematogenic effect in a carrageenan-induced paw edema model [23,24]. Palmatine showed anti-hyperalgesic effects against diabetic neuropathy (mechanical and thermal hyperalgesia) [30] and trigeminal neuralgia models (mechanical hyperalgesia) in rats. Our results showed that EMAS (100 mg/kg) and palmatine (1 mg/kg) had no activity in the first phase (Figure 4A), but in the second phase (inflammatory phase) (Figure 4B), nociception was antagonized by palmatine. Furthermore, EMAS and palmatine prevented mechanical hyperalgesia (Figure 4C) and cold sensitivity (Figure 4D). The present study was the first to demonstrate palmatine anti-nociceptive properties in the formalin model, which could partially explain the popular analgesic use of *A. squamosa*.

The extract was chemically analyzed and revealed the presence of palmatine, lirio-denine, and anonaine. Palmatine is an alkaloid present in several medicinal plants and has a wide range of pharmacological activities already described in the literature [34], including anticancer [23,38,55], antioxidant [56,57], anti-inflammatory [31,32], neuroprotective [29,56,58], antibacterial [59,60], antiviral [59,61], and hypolipidemic properties [26,62]. Previous studies have demonstrated the anti-inflammatory activity of liriodenine. Liriodenine reduced NO production by macrophages obtained from lipopolysaccharide (LPS)-induced peritonitis in rats [63], interacted with the COX-2 binding site and inhibited COX-2 in silico [64], decreased cell proliferation in the rat synovial cavity, and reduced splenocyte proliferation in vitro [63]. Anonaine has not been evaluated for inflammatory or analgesic effects. The effective dose of EMAS was 100 mg/kg, and the dose of 1 mg/kg of palmatine was selected to be tested in zymosan and formalin models, since palmatine comprises approximately 1% of EMAS (performed by validated quantitative HPLC-ESI method). Both EMAS (100 mg/kg) and palmatine (1 mg/kg) had efficacy in all the models tested in the present work. The present study was the first to show that palmatine is the main compound found in EMAS, responsible for anti-nociceptive and anti-arthritic effects.

Given the effects of palmatine on pain and inflammation, this study evaluated the involvement of the TNF pathway in the anti-hyperalgesic effect of this substance. TNF is a potent pro-inflammatory cytokine that plays an important role in pathological spinal pain [65,66]. This cytokine acts as a neuromodulator in the dorsal horn of the spinal cord [65] and regulates both inflammatory and neuropathic pain [66,67,68]. In this study, palmatine was observed to inhibit TNF-induced mechanical hyperalgesia, which is consistent with previous studies showing that palmatine antagonized TNF-α and IL-1β in neuropathy models [30,36]. The anti-hyperalgesic effect of palmatine was associated with the up-regulation of calcitonin gene-related peptide (CGRP) expression, a neurotransmitter with an important role in pain transmission [36]. In diabetic neuropathy, palmatine decreased phosphorylation of the ERK1/2 pathway, which contributed to a reduction in the expression of P2X7, a receptor with an important role in the development of pain and depression [30]. Palmatine showed efficacy in the TNF model, but the result of this test does not exclude that palmatine antagonizes other inflammatory/nociceptive pathways.

Considering the role of leukocytes in the regulation of inflammation and, consequently, inflammatory pain, the viability of leukocytes treated with palmatine and EMAS was evaluated by the in vitro MTT test. Although no significant cytotoxicity was observed, it is interesting to note that studies have been published linking palmatine to the induction of DNA modification [34]. In addition, palmatine has a complex effect on metabolic enzymes in the liver, with glucuronidation and sulfation being the main metabolic pathways [34]. In in vitro tests, palmatine showed cytotoxic potential in A431 cells (a human skin epithelial carcinoma cell line) [69], while in cardiomyocyte cell culture, this substance showed cardiotoxic potential [70]. In human hepatoma cells and in normal liver cells, this substance did not induce cytotoxic cells [71]. In addition to palmatine, the aqueous extract of the leaves of *A. squamosa* showed no signs of toxicity at a dose of 2000 mg/kg. There were, however, signs of intoxication such as salivation, convulsion, excitation, and motility 48 h after administration of the extract at a dose of 5000 mg/kg [72].

This study has some limitations, for example, palmatine and EMAS were not evaluated in toxicity models. Another limitation is the use of only mice and a few doses of EMAS and palmatine in the experimental models, but the ethics of the use of animal experimentation limits the use of animals.

## 4. Materials and Methods

### 4.1. Plant Material, EMAS Acquisition, and Isolation/Chemical Analysis of Palmatine

The leaves of *A. squamosa* were collected from a specimen grown at the Biology Institute of Unicamp (22°49′10.1″ S 47°04′16.3″ W) and registered in the National System for the Management of Genetic Heritage and Associated Traditional Knowledge (Access Register No. A709776). The collected plant raw material was stabilized and dried in a circulating air oven at 40 °C (up to seven days) and then pulverized in a knife mill. In the present work, a methanolic extract of *A. squamosa* leaves was prepared due to the selectivity of the methanol solvent for the extraction of protoberberine alkaloids, the focus of this study, such as palmatine. The obtained powder (1000 g) was successively extracted (maceration) with n-hexane, followed by MeOH, in the ration 1:20 (powder/extracting liquid, *v/v*). The extraction was performed first with hexane (clean up), and then the same material, after treatment with hexane, underwent the extraction with MeOH to extract the alkaloids. After evaporation of the extracted liquid in an evaporator under reduced pressure, the crude hexane (121.5 g) and methanol (EMAS, 72.3 g) extracts were obtained. TLC (thin layer chromatography) and UHPLC-ESI-MS (ultra high-performance liquid chromatography–electrospray ionization tandem mass spectrometry) investigations indicated the presence of alkaloids in the EMAS extract. An aliquot of EMAS (2.0 g) was initially subjected to acid–base extraction according to COSTA et al. (2010) [37] for the isolation of alkaloid constituents, and to give the CH_2_Cl_2_ alkaloid fraction (0.25 g) and the CH_2_Cl_2_ neutral fraction (1.75 g). The alkaloid fraction (0.20 g) was subjected to 10% NaHCO_3_-treated silica gel column chromatography eluted with the following gradient systems: petroleum ether–CH_2_Cl_2_ from 100:0 to 10:90, followed by CH_2_Cl_2_-EtOAc from 100:0 to 10:90, and EtOAc-MeOH from 100:0 to 50:50. The eluted fractions (35 fractions of 30 mL each) were evaluated and pooled according to TLC analysis to afford 15 fractions. Fraction 12 (25.0 mg) was purified by preparative TLC eluted with CH_2_Cl_2_-MeOH (96:04) to give the alkaloid palmatine (20.0 mg). All solvents used in these procedures were rota-evaporated, aiming to ensure their full removal from the product. The isolated protoberberine alkaloid palmatine was identified using spectroscopic (UV, IR, 1D, and 2D NMR) and mass spectrometric (ESI-MS) methods. For the identification analysis by UHPLC-ESI-MS/MS of the dried crude extract (EMAS), the fractions obtained from the chromatographic columns and the standard compounds isolated by our research group were dissolved in methanol (at 1.0 mg/mL) and filtered (0.22 μm) to proceed with the analysis. The chemical profile and analysis of the presence of alkaloids in the extracts was performed by ESI-MS/MS with the crude extracts and fractions. The confirmation of the presence of alkaloids in the samples was performed by direct injection into the Quadrupolar Mass Spectrometer—Micromass (Acquity model from Micromass/Waters) by electrospray in positive mode. The constituents were identified by comparing their ESI-MS/MS fragmentation spectra with the spectra of the standard compound previously isolated by our research group and with data from the literature. All chromatographic analysis of the samples was performed was performed using an Acquity UHPLC chromatograph coupled to an Acquity TQD mass spectrometer (Micromass-Waters, Manchester, UK), with ESI source, using a Waters Acquity C_18_ BEH column (2.1 mm × 50 mm × 1.7 µm particle size). The analysis conditions were as follows: solvent A (Milli-Q water and formic acid 0.1%) and solvent B (methanol). The flow rate was 0.2 mL/min for the entire run time of 7.0 min, with an elution gradient that started with 20% methanol and went up to 100% at minute 5, 100% from 5.01 to 5.50 min, and finally returning to the initial situation. The detection was performed in positive mode ionization under 3.00 kV of capillary voltage, 30.00 V at cone, 130 °C source temperature, 250 °C desolvation temperature, and an injection volume of 5 μL. The analyzed masses were in the range of 100 to 800 *m/z*. Finally, the constituents were identified by UHPLC-MS/MS analysis, comparing the mass spectra, the retention time, and the fragmentation profile (MS/MS) of the standard compound spectra previously isolated by our research group with the fragmentation spectra of the crude extract and fractions. Collision-Induced Dissociation (CID) was performed to identify the ions, and the MS/MS spectra were compared with the fragmentation pattern of the substances annonaine, asimilobine, liriodenine, lysicamine, isomoschatoline, o-methylisomoschatoline, coreximine, isocoreximine, reticuline, 9-methoxy-isomoschatoline, and palmatine, and with data found in the literature (collision energy 30 V). For the quantification of palmatine in the EMAS extract, the palmatine standard (Sigma-Aldrich, St. Louis, MA, USA) at concentrations of 0.010 to 1.0 mg/mL was prepared and analyzed with the HPLC-ESI-MS method described before. An accurately weighed amount of EMAS was dissolved in methanol and analyzed with the same chromatographic conditions as used for palmatine. After chromatographic analysis by UPLC-MS (n = 3), the regression line was obtained by plotting the concentrations of alkaloid on the abscissa axis and the sample area on the ordinate axis. Calibration graphs were plotted, showing a linear relationship between concentrations versus peak areas for the palmatine reference compound. The regression equation curve obtained for the palmatine was y = 138,151,203x − 112,281, R^2^ = 0.9887. The attribution of the chromatographic peak was based on the retention time of the single compound and confirmed by analysis in comparison with the isolated standard. Under our working conditions, the mean retention time for palmatine was 2.44 min. The concentration was calculated from the experimental peak area by analytical interpolation in standard calibration line. The limit of quantification (LOQ) for palmatine was 0.010 μg/mL. The relative standard deviation (%RSD) was 0.15%, calculated as the mean of the three replications

### 4.2. Reagents

Zymosan, carrageenan, Bradford solution, methylthiazolidiphenyl-tetrazolium bromide, and RPMI medium were purchased from Sigma-Aldrich (Saint Louis, MO, USA). Tumor necrosis factor was obtained from Abcam (Cambridge, UK). Phosphate-buffered saline, ethylene diamine tetra acetic acid, acetone, and dimethyl sulfoxide were acquired from Vetec (Rio de Janeir, Brazil). Turk’s solution was purchased from Newprov (Pinhais, Brazil). Ketamine and xylazine were obtained from Syntec (Santana de Paraíba, Brazil). Formalin was acquired from Proquimicos (Rio de Janeiro, Brazil). All reagents used for this work have a high analytical grade.

### 4.3. Animals, Dissolution of EMAS and Palmatine, and Description of the Doses Used in the In Vivo and In Vitro Models

Female and male Swiss mice (28–32 g) were provided by the central biotherium of the Federal University of Grande Dourados (UFGD). Animals were housed in polypropylene boxes in the biotherium of the Health Sciences College under the following conditions: 22 °C, in the presence of a 12-hour light/dark cycle, with food and water ad libitum. The experiments were conducted at the UFGD. The experiments were performed after approval by the UFGD Animal Use Ethics Committee (13.2020).

EMAS and palmatine were dissolved in saline solution (0.9%), which was the vehicle used to treat the animals, and the dose was calculated according to the weight of the animals.

The oral inhibitory effect of EMAS (30, 100 and 300 mg/kg) and palmatine (0.3, 1, or 3 mg/kg) was investigated to evaluate if there was change in the response at different doses in the carrageenan-induced pleurisy model.

The effectives doses of EMAS were 100 and 300 mg/kg, and of palmatine were 1 and 3 mg/kg of palmatine, and a dose of 100 mg of extract and 1 mg/kg of the pure substance were selected to test in the zymosan and formalin models. As mentioned before, the yield of palmatine in EMAS was 1%, and the dose of 100 mg/kg of EMAS was equivalent to 1 mg/kg of palmatine. In addition, prednisolone (3 mg/kg, orally) was used as a positive control drug.

### 4.4. EMAS and Palmatine Test in Carrageenan-Induced Pleurisy

Female Swiss mice were divided into groups (9 groups with n = 5) and treated orally with vehicle (naive and control groups), EMAS (30, 100 or 300 mg/kg), palmatine (0.3, 1, or 3 mg/kg), and prednisolone (3 mg/kg).

Inflammation in the pleural cavity was induced by a 100 µL injection of carrageenan solution (1% diluted in sterile saline) one hour after previous oral administration [73]. Animals in the naive group received similar volume injections of 0.9% saline. Four hours after the induction of pleurisy, animals were submitted to a euthanasia protocol (ketamine—300 mg/kg, intraperitoneal (i.p.) + xylazine—30 mg/kg, i.p.). The thoracic cavity was opened, and the exudate was collected by washing the cavity in 1000 µL of phosphate-buffered saline/ethylene diamine tetra acetic acid (PBS/EDTA) solution. Part of the obtained sample was diluted in Turk’s fluid for leukocyte count using a Neubauer chamber, while another part was used for nitrite dosage (a final product of the nitric oxide oxidation pathway) by the Griess method.

### 4.5. EMAS and Palmatine Test on Zymosan-Induced Joint Inflammation

Female Swiss mice were divided into four groups and treated with vehicle (control), palmatine (1 mg/kg), EMAS (100 mg/kg), or prednisolone (3 mg/kg—positive control drug). One hour later, inflammation was induced by an intra-articular (i.a.) injection of zymosan (200 μg/cavity in 10 μL sterile saline solution) into the left knee junction through the suprapatellar ligament [74]. Edema (digital micrometer) and mechanical hyperalgesia (electronic Von Frey) were measured 4 and 6 h after zymosan injection. Six hours after the induction of inflammation, the animals were euthanized (with a dose of 300 mg/kg of ketamine and 30 mg/kg of xylazine, by intraperitoneal route (i.p.). The inflamed joint was surgically exposed and washed with 10 μL of PBS containing EDTA (10 mM). Subsequently, 40 μL of this solution of PBS/EDTA was added to the collected exudate. Part of the sample was diluted in Turk’s solution for leukocyte count in a Neubauer chamber.

### 4.6. EMAS and Palmatine Test on Formalin-Induced Nociception

Male Swiss mice were distributed into 3 groups (n = 5) and treated orally with vehicle (control), palmatine (1 mg/kg), or EMAS (100 mg/kg). One hour after administration, 1% formalin (30 μL/animal) was injected into the right hind paw of the animals (intraplantar route). The mice were placed individually in glass funnels and observed for nociceptive behavior (licking and biting) during two phases (0–5 min initially and a second phase at 15–30 min) [54,75].

Subsequently, 30 μL of acetone was applied to the surface of the injected paw, and the times the animal lifted its paw in the first 20 s were counted. In addition, mechanical hyperalgesia was analyzed using the electronic Von Frey, and the edema volume was measured using a digital plethysmometer.

### 4.7. Palmatine Test on TNF-Induced Hyperalgesia

The TNF-induced hyperalgesia test was performed according to Aquino et al. (2015) [76] to verify whether palmatine has a direct effect on mechanical hyperalgesia. Prior to the intraplantar administration of palmatine, the baseline mechanical score of the right hind paw of all animals was determined using a digital Von Frey algesimeter.

The animals were then distributed into 3 groups (n = 5) and intraplantarly received 30 µL of the following solutions (right hind paw): vehicle (control) and palmatine solution (3 and 30 μg/paw). After 30 min, an intraplantar injection of 20 μL of TNF (100 pg) was made into the same previously treated paw. Three and four hours after the induction of inflammation by TNF, the mechanical hyperalgesia of the right hind paw was measured by the digital Von Frey algesimeter and the animals were euthanized with dose of 300 mg/kg of ketamine and 30 mg/kg of xylazine (i.p.) after the last measurement of mechanical hyperalgesia.

### 4.8. Analysis of EMAS and Palmatine on Leukocyte Viability by Methylthiazolidiphenyl-Tetrazolium (MTT) Bromide Test

The MTT assay is based on applying the mitochondrial enzyme reduction in tetrazolium dye to detect and determine cell viability. This assay was performed as previously described by Silva-Filho et al. [77]. The leukocytes were obtained from the peritoneal cavity of male Swiss mice 4 h after an intraperitoneal injection of zymosan (1 mg/cavity). The cells numbers were plated at 5 × 10^5^ cells/well in 100 µL RPMI medium (supplemented with 10% of fetal bovine serum and penicillin 100 U/mL + streptomycin 100 μg/mL) into 96-well plates. The cells were exposed to EMAS or palmatine at concentrations of 10, 30, 90 μg/mL, or vehicle, and incubated by 90 min (37 °C/5% CO_2_). Then, 10 µL of MTT solution (5 mg/mL) was added to each well. After 2 h of incubation (37 °C/5% CO_2_), the supernatant was removed, and 100 µL of dimethyl sulfoxide was added to each well. The plate was incubated for another 10 min and the reading was performed by an ELISA reader at 540 nm. The values of the blank wells were subtracted from each well of the treated and control cells. The percentage of feasibility was determined by the following formula:

$$\%\mathrm{Viable}\;\mathrm{cells}=\frac{\left(\mathrm{absorbance}\;\mathrm{of}\;\mathrm{the}\;\mathrm{treates}\;\mathrm{cells}-\mathrm{Absorbance}\;\mathrm{of}\;\mathrm{the}\;\mathrm{blank}\right)}{\left(\mathrm{Absorbance}\;\mathrm{of}\;\mathrm{the}\;\mathrm{control}-\mathrm{Abosorbance}\;\mathrm{of}\;\mathrm{the}\;\mathrm{blank}\right)}\times 100$$

### 4.9. Statistical Analysis

Data are presented as the mean ± standard error (SEM). Determination of significant differences among groups was performed using a one-way analysis of variance (ANOVA) followed by a Tukey post hoc test (GraphPad Prism Software). The percentage of inhibition was calculated from the control group. Differences were considered significant when *p* < 0.05.

## 5. Conclusions

This study demonstrated the presence of palmatine and other compounds in the leaves of *A. squamosa*. The analgesic, anti-inflammatory, and anti-arthritic potential effects of EMAS and palmatine were demonstrated in inflammation, arthritis, and nociception experimental models. Taken together, the data suggest that EMAS and palmatine may have therapeutic potential in arthritis and pain disorders. One of the mechanisms of action of palmatine may be the TNF pathway.

## Figures and Tables

**Table 1:** UHPLC-MS analysis of the alkaloids (confirmed by a comparison with a standard sample) in the methanolic extract of *A. squamosa* leaves (EMAS).

Analyzed Compounds ^a^	Masses Calculated	Positive Ion Mode [M + H]^+^ (*m/z*)	Retention Time (min)	EMAS
Anonaine	265	266	2.86	+
Asimilobine	267	268	2.19	-
Liriodenine	275	276	3.00	+
Lysicamine	291	292	3.63	-
Isomoschatoline	307	308	4.01	-
O-methyl-isomoschatoline	321	322	4.45	-
Coreximine	327	328	1.36	-
Isocoreximine	327	328	1.36	-
Reticuline	329	330	1.61	-
9-Methoxy-isomoscatoline	337	338	4.18	-
Palmatine	352	353	2.44	+

+: compound detected; -: compound not detected. ^a^ Standard sample.

## Data Availability

All data are available in this publication.

## Supplementary Materials

The following supporting information can be downloaded at https://www.mdpi.com/article/10.3390/ph17101331/s1, Table S1. Palmatine ^1^H and ^13^C-NMR and gHMBC data. Figure S1: Palmatine molecular structure. Figure S2: ^1^H NMR spectra of palmatine (400 MHz, CDCl_3_); Figure S3: ^1^H-^13^C correlation map from HSQC NMR experiment of palmatine (400 MHz for ^1^H e 100 MHz for ^13^C, CDCl_3_); Figure S4: ^1^H-^13^C long-range correlation map from HMBC NMR experiment of palmatine (400 MHz for ^1^H e 100 MHz for ^13^C, CDCl_3_); Figure S5: ESI(+)-MS/MS. Fragmentation of the alkaloid palmatine (*m*/*z* 352).
