# Supplementary material for: Analgesic and Anti-Arthritic Potential of Methanolic Extract and Palmatine Obtained from Annona squamosa Leaves
Source: Pharmaceuticals (Basel). 2024 Oct 5;17(10):1331. doi: 10.3390/ph17101331 (PMC11510468; doi:10.3390/ph17101331)
Supplement: Supplementary file 1 [file pharmaceuticals-17-01331-s001.zip › pharmaceuticals-3152442-supplementary.pdf]

pharmaceuticals

MDPI

Supplementary Material

Analgesic and anti-arthritic potential of methanolic extract and palmatine obtained from *Annona squamosa* leaves

Caren Naomi Aguero Ito [1], Elisangela dos Santos [1], Natália de Matos Balsalobre [1], Lucas Luiz Machado [2], Saulo Euclides Silva-Filho [2]*, Taíse Fonseca Pedroso [3], Caroline Caramano de Lourenço [3], Rodrigo Juliano Oliveira [4], Arielle Cristina Arena [5], Marcos José Salvador [3], Cândida Aparecida Leite Kassuya [1]*

[1] Health Sciences College, Federal University of Grande Dourados (UFGD), Dourados, Zip code 79.804-970, Mato Grosso do Sul (MS), Brazil; itonaomi@live.com (C.N.A.I.); elisangelaprocopiosan@gmail.com (E.d.S.); nataliabalsalobre@hotmail.com (N.M.B.); candida2005@gmail.com (C.A.L.K.)

[2] Pharmaceutical Sciences, Food and Nutrition College, Federal University of Mato Grosso do Sul (UFMS), Campo Grande, Zip code 79070-900, MS, Brazil; lucaslmachado11@gmail.com (L.L.M.); saulo.e@ufms.br (S.E.S.-F.)

[3] Institute of Biology, Department of Plant Biology, University of Campinas (UNICAMP), Campinas, Zip code 13.083-862, São Paulo, Brazil; taisefpedroso@gmail.com (T.F.P.); ca_jau@hotmail.com (C.C.d.L.); marcosjs@unicamp.br (M.J.S.)

[4] Stem Cell, Cell Therapy and Toxicological Genetics Research Centre (CeTroGen), Medical School, Federal University of Mato Grosso do Sul, Campo Grande, Mato Grosso do Sul 79080-190, Brazil; rjo.rodrigojulianooliveira@gmail.com (R.J.O)

[5] Institute of Biosciences of Botucatu, Department of Structural and Functional Biology, São Paulo State University (UNESP), Botucatu, Zip code 18.618-970, SP. Brazil; arielle.arena@unesp.br (A.C.A.)

* Correspondence: candida2005@gmail.com (C.A.L.K.); saulo.e@ufms.br (S.E.S.-F.)

Palmatine NMR data

Palmatine (Figure 1) ^{1}H-NMR, gHMBC (heteronuclear multiple Bond correlation) and gHSQC (heteronuclear single quantum coherence) spectra were obtained in a Bruker 400 equipment. $CDCl_3$ was employed as solvent and Tetramethylsilane (TMS) as internal reference. The analysis gHMBC and gHSQC confirmed the correlation between hydrogen and carbon atoms as shown in Table S1 (Figures S2, S3 and S4) and presents the ^{1}H and ^{13}C-NMR values that were shown to be compatible with the tetrahydroprotoberberine quaternary alkaloid known as palmatine by comparisons with the data described in the literature (CHIA et al., 1998; WAFO et al., 1999; GRYCOVÁ et al., 2007; COSTA et al., 2009; COSTA et al., 2010; COSTA et al., 2013). Furthermore, by analyzing the ESI-MS/MS mass spectrum obtained in positive ion mode by direct injection, an ion with $[M+H]^+$ = 353 m/z was verified, with a fragmentation profile also compatible with the alkaloid palmatine (Figure S5).

The sample identified as Palmatine appeared as a yellowish needle-shaped solid compatible with the molecular formula $C_{21}H_{22}NO_4$, inferred based on the NMR 1D and 2D spectra (Figures S2, S3 and S4, Table S1) and confirmed by analysis of the ESI-MS/MS mass spectrum obtained in positive ion mode, which showed m/z $[M+H]^+$ = 353 (Figure 5). In the ^{1}H-NMR spectrum (400 MHz, $CDCl_3$), two hydrogens signals were observed at δ 9.79 (1H, s) and δ 8.69 (1H, s), typical of a berberine skeleton, which were attributed to H-8 and H-13. Two signals of coupled methylene groups, one at δ 3.32 (2H, t, J = 6.3 Hz) and the other relatively displaced (unshielded) at δ 4.98 (2H, t, J = 6.3 Hz), this effect being caused by the presence of a quaternary nitrogen, these signals being characteristic of H-5 and H-6, respectively, compatible with hydrogen signals from the protoberberine skeleton (CHIA et al., 1998; WAFO et al., 1999; GRYCOVÁ et al., 2007; COSTA et al., 2009; COSTA et al., 2010; COSTA et al., 2013). Four typical hydrogens signals of methoxylic group were also observed at δ 4.00, δ 4.06, δ 4.12 and δ 4.25, all with integration for three hydrogens, two singlets at δ 7.56 and δ 6.96 attributed to two isolated aromatic hydrogens

located in the para position, characteristic of a 2,3,5-6-tetrasubstituted benzene ring, and a superimposed aromatic hydrogen signal at δ 8.02 with integration for two hydrogens (Table S1). The analysis *g*HMBC and *g*HSQC confirmed the correlation between hydrogen and carbon atoms as shown in Table 1. The analysis of the NMR, *g*HSQC and *g*HMBC spectra revealed the presence of 21 carbons, 15 of which were aromatic between δ 152.86 and δ 108.77, four methoxylic at δ 62.35, δ 57.32, δ 56.67 and δ 56.50 and two methylene at δ 27.35 and δ 56.79 typical of C-5 and C-6 (Table 1). The presence of two hydrogenated aromatic carbon signals at 145.15 (H-8) and 120.66 (H-13) confirms the presence of the berberine skeleton (CHIA et al., 1998; WAFO et al., 1999; GRYCOVÁ et al., 2007). The correct positions of the aromatic hydrogens were determined by analyzing the HSQC and HMBC contour maps with the vicinal and aliphatic aromatic carbons. The signals of the hydrogens at δ 7.56 attached to carbon δ 108.77 and δ 6.96 attached to carbon δ 111.37 were assigned to H-1 and H-4, respectively, thus defining the para system in ring A, and the signal at δ 8.02 (2H) was assigned to H-11/H-12, thus defining the ortho system superimposed on ring D. The existence of the correlation of the signal at δ 6.96 (H-4) with the signal at J3 of the carbon at δ 27.35 (C-5), and δ 7.56 (H-1) with the signal at J3 of the carbon at δ 138.76 (C-13a) indicated unambiguously the position of the aromatic hydrogens. Consequently, the superimposed ortho system was defined in ring D by correlating the signal at δ 8.02 linked to carbons 127.39 (C-11) and 123.60 (C-12) at J3 with the signals from carbons at δ 122.39 (C-8), δ 134.24 (C-12a) and δ 120.66 (C-13), thus elucidating this part of the molecule (CHIA et al., 1998; WAFO et al., 1999; GRYCOVÁ et al., 2007; COSTA et al., 2009; COSTA et al., 2010; COSTA et al., 2013).

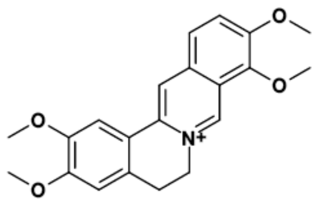

Figure S1. Palmatine molecular structure.

Table S1. Palmatine ^{1}H and ^{13}C-NMR and *g*HMBC data.

Position	^{1}H (δ) (mult.,*J* in Hz)[a]	^{13}C (δ)[a,b]	*g*HMBC (^{1}H-^{13}C)[c]
1	7.56 (1H, *s*)	108.77 d	2, 3, 4, 4a,13a and 13b
2		150.01 s	
3		152.86 s	
4	6.96 (1H, *s*)	111.37 d	1, 2, 3, 4a, 5, 13a and 13b
4a		128.64 s	
5	3.32 (2H, *t*, 6.3)	27.35 t	4, 4a, 5 and 13b
6	4.98 (2H, *t*, 6.3)	56.79 t	4a, 5, 8 and 13b
8	9.79 (1H, *s*)	145.15 d	6, 8a, 9, 12a, 13 and 13a
8a		122.39 s	
9		144.33 s	
10		150.87 s	
11	8.02 (1H, *s*)	127.39 d	9, 12 and 12a
12	8.02 (1H, *s*)	123.60 d	8a, 10, 12a and 13
12a		134.24 s	
13	8.69 (1H, *s*)	120.66 d	8a, 12, 13a and 13b
13a		138.76 s	
13b		119.26 s	
2-OCH_3	4.06 (3H, *s*)	56.67 q	2
3-OCH_3	4.00 (3H, *s*)	56.50 q	3
9-OCH_3	4.25 (3H, *s*)	62.35 q	9
10-OCH_3	4.12 (3H, *s*)	57.32 q	10

[a] Analysis were performed in a Bruker 400 equipment and the experiments were acquired with tetramethylsilane (TMS) as internal reference standard (0.00 ppm) in $CDCl_3$ at 400 MHz for ^{1}H NMR and 100 MHz for ^{13}C NMR. [b] Multiplicities determined by gHSQC spectra. [c] Carbon atoms that correlate with its respectively hydrogens. (*δ*) chemical shift (ppm). (*J*) coupling constant.

NMR data for palmatine

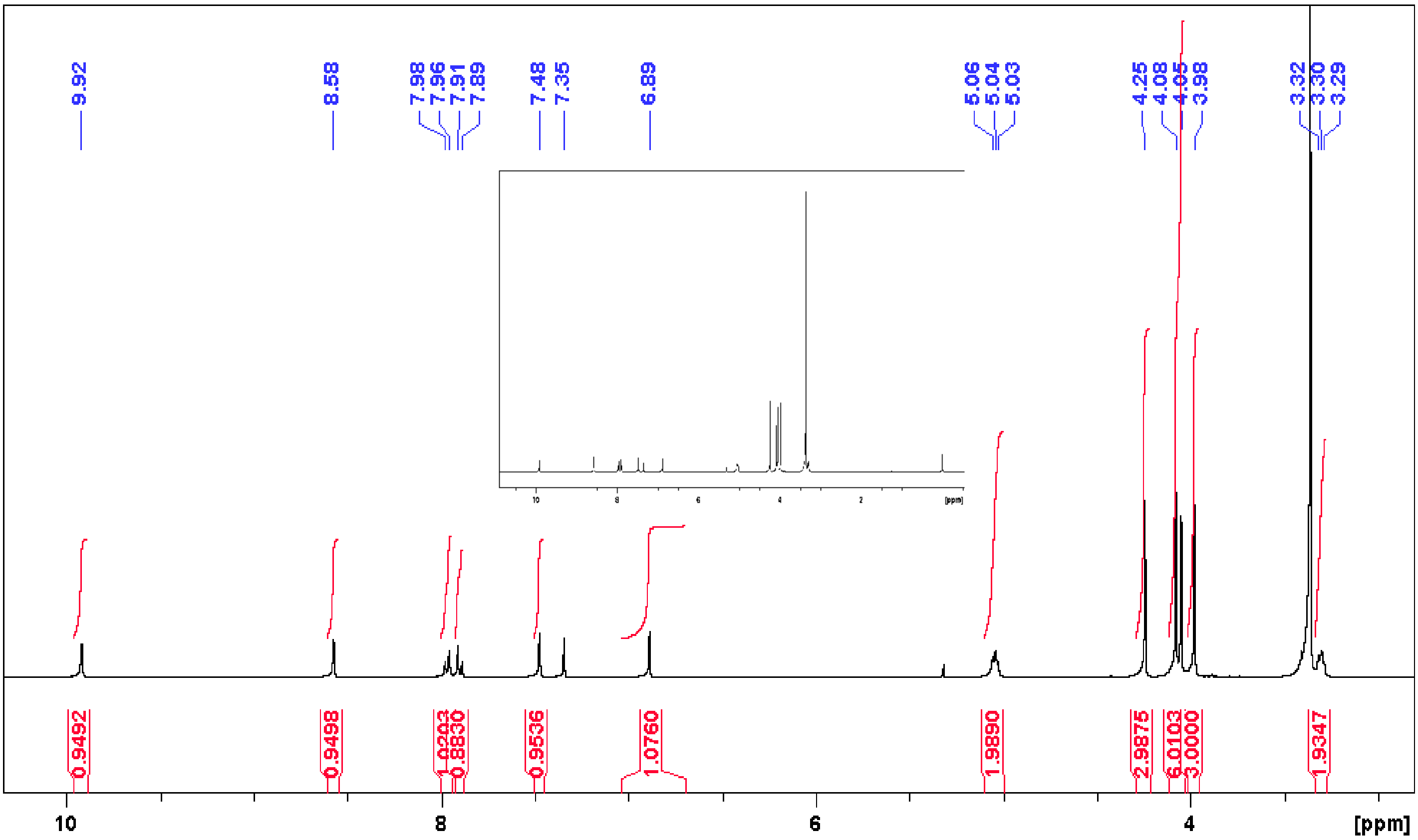

Figure S2. ^{1}H NMR spectra of palmatine (400 MHz, $CDCl_3$).

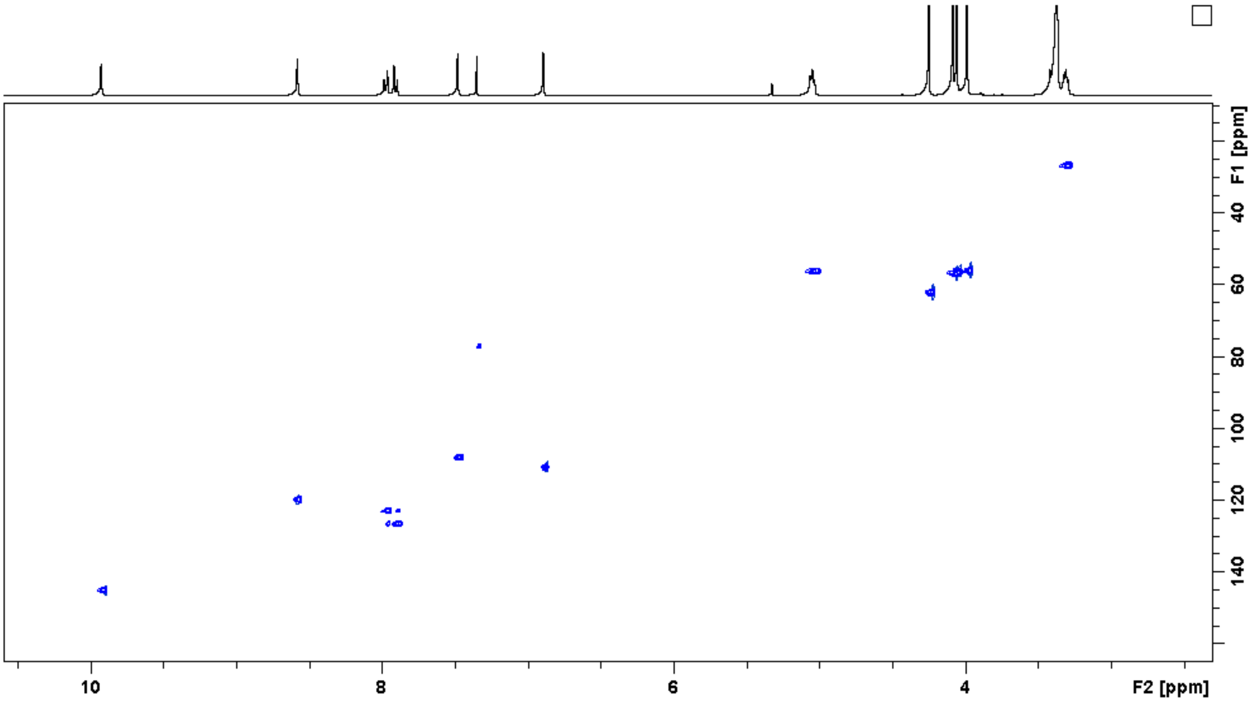

Figure S3. ^{1}H-^{13}C correlation map from HSQC NMR experiment of palmatine (400 MHz for ^{1}H e 100 MHz for ^{13}C, $CDCl_3$).

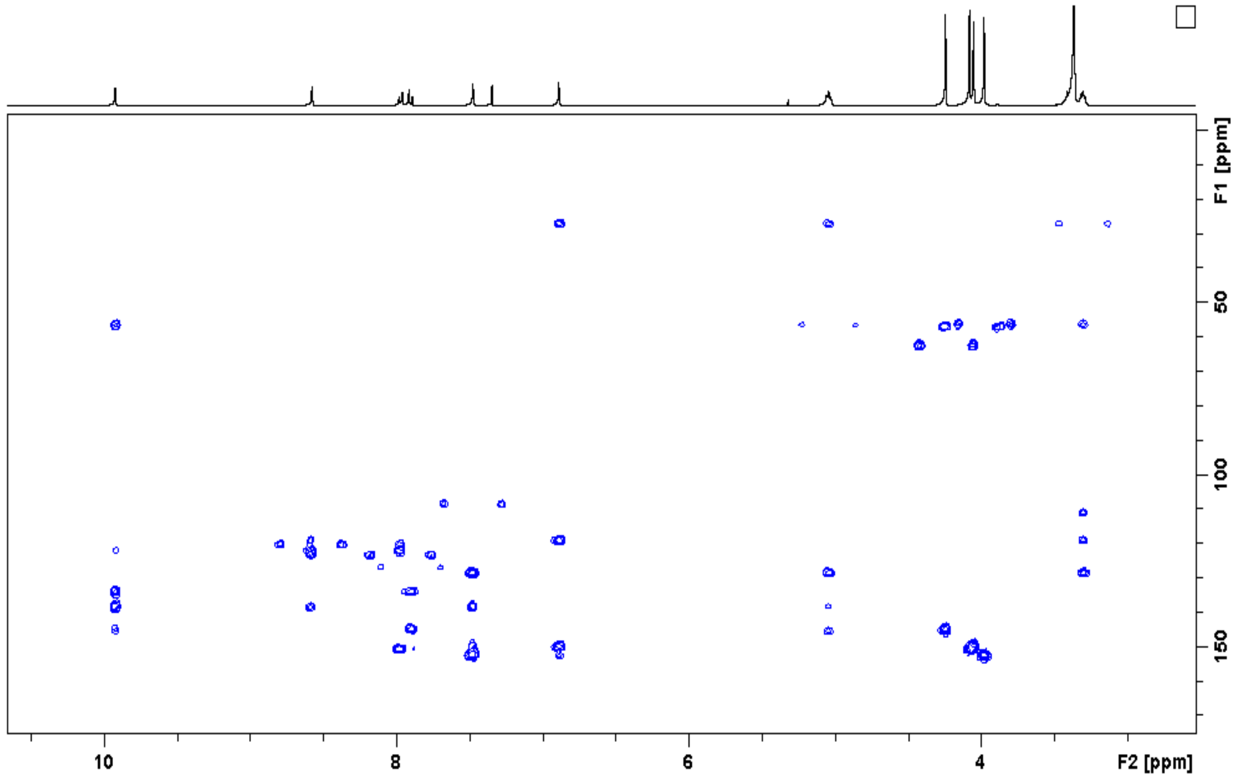

Figure S4. ^{1}H-^{13}C long-range correlation map from HMBC NMR experiment of palmatine (400 MHz for ^{1}H e 100 MHz for ^{13}C, $CDCl_3$).

Palmatine ESI-MS data

Palmatine presented as a yellowish needle-shaped solid with a molecular mass equal to 352.0 g.Mol^{-1}, confirmed by analysis of the ESI-MS/MS mass spectrum obtained in positive ion mode, which showed *m*/*z* $[M+H]^{+}$ = 353.2 and a fragmentation profile compatible with the alkaloid palmatine (Figure 5). ESI-MS analysis were performed using methanol as solvent.

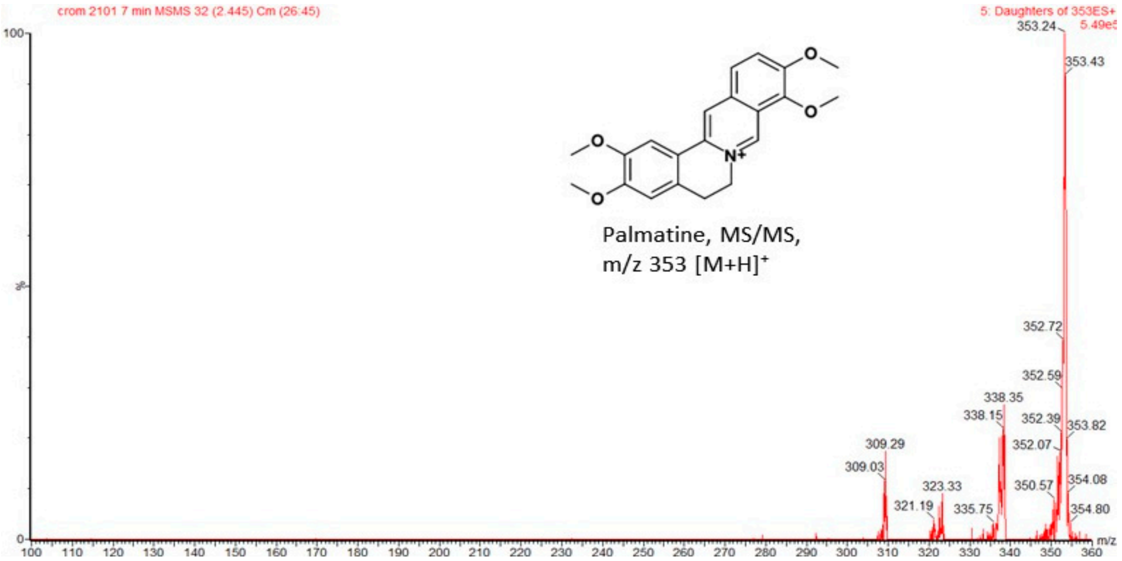

Figure S5. ESI(+)-MS/MS. Fragmentation of the alkaloid palmatine (m/z 352).

References

Chia, Y. C.; Chang, F. R.; Ming, C.M.; Wu, Y.C. Protoberberine Alkaloids from Fissistigma balansae. *Phytochemistry*, 1998, 48, 367-369.

Grycová, L.; Dostal, J.; Marek, R. Quaternary Protoberberine Alkaloids. *Phytochemistry*, 2007, 68, 150-175.

Wafo, P.; Nyasse, B.; Fontaine, C. A. 7,8-dihydro-8-hydroxypalmatine from *Enantia chlorantha*. *Phytochemistry*, 1999, 50, 279-281.

Costa, E. V.; Pinheiro, M. L. B.; Silva, J. R. D. A.; et al. Antimicrobial and Antileishmanial Aactivity of Essential Oil from the Leaves of *Annona foetida* (Annonaceae). *Quimica Nova*, 2009. 32, 78–81.

Costa, E.V.; Pinheiro, M.L.B.; Barison, A.; Campos, F.R.; Salvador, M.J.; Maia, B.H.L.N.S.; Cabral, E.C.; Eberlin, M.N. Alkaloids from the Bark of *Guatteria hispida* and Their Evaluation as Antioxidant and Antimicrobial Agents. *J. Nat. Prod.* 2010, 73, 1180–1183, doi:10.1021/np100013r.

Costa, E. V.; Da Cruz, P. E. O.; Pinheiro, M. L. B.; Marques, F. A.; Ruiz, A. L .T. G.; Marchetti, G. M.; Carvalho, J. E.; Barison, A.; Maia, B. H. L. N. S. Aporphine and Tetrahydroprotoberberine Alkaloids from the Leaves of *Guatteria friesiana* (Annonaceae) and their Cytotoxic Activities. *Journal Brazilian Chemical Society*, 2013, 24, 788-796.
